# Serum Sickness-Like Reaction: A Narrative Review of Epidemiology, Immunopathogenesis, Diagnostic Challenges, and Therapeutic Approaches

**DOI:** 10.3390/clinpract15100178

**Published:** 2025-09-26

**Authors:** Christodoulos Chatzigrigoriadis, Emmanouil Koufopoulos, Prodromos Avramidis, Ioannis Erginousakis, Vasiliki Karakoida, Theofanis Papadopoulos, Despoina Sperdouli, Myrsini-Eirini Tachliabouri, Kyriakos Vilanakis, Dimitrios Zampounidis, Vasiliki Michou, Panagiotis Eskitzis, Panagis Galiatsatos, Lazaros Lavasidis, Doxakis Anestakis

**Affiliations:** 1 School of Medicine, University of Patras, 26504 Patras, Greece; up1084142@ac.upatras.gr (C.C.); up1084128@ac.upatras.gr (E.K.); up1084139@ac.upatras.gr (P.A.); up1084168@ac.upatras.gr (I.E.); bkarakoida@gmail.com (V.K.); chem3793@ac.upatras.gr (M.-E.T.); up1089614@ac.upatras.gr (K.V.); up1084171@ac.upatras.gr (D.Z.); 2 Department of Nursing, International Hellenic University, 57001 Thessaloniki, Greece; theopapa36@ihu.gr; 3 Department of Internal Medicine, General Hospital of Thebes, 15231 Thebes, Greece; 4 School of Medicine, National Kapodistrian University of Athens, 10679 Athens, Greece; deppysper@yahoo.com; 5 Department of Midwifery, School of Healthcare Sciences, University of Western Macedonia, Keptse, 50200 Ptolemaida, Greece; peskitzis@uowm.gr (P.E.); llavasidis@uowm.gr (L.L.); 6 Department of Internal Medicine, Johns Hopkins University School of Medicine, Baltimore, MD 21224, USA; pgaliat1@jhmi.edu; 7 Department of Pathology and Forensic Medicine, Medical School, University of Cyprus, 1678 Nicosia, Cyprus; anestaki@auth.gr; 8 Laboratory of Autopsy Pathology, Forensic Service of Thessaloniki, Ministry of Justice, 54124 Thessaloniki, Greece

**Keywords:** serum sickness-like reaction, drug hypersensitivity, diagnosis, treatment

## Abstract

Serum sickness-like reaction (SSLR) is a rare immune-mediated condition that typically affects the skin and joints after exposure to certain drugs, infections, or vaccines. Although it shares clinical similarities with serum sickness (SS), SSLR differs in its underlying mechanisms, histopathology, and causes. Despite its generally benign and self-limiting nature, SSLR is frequently misdiagnosed and may lead to unnecessary hospitalization. This narrative review summarizes current knowledge on epidemiology, pathophysiology, clinical features, diagnosis, treatment, and long-term considerations related to SSLR. The condition is most often associated with antibiotics, monoclonal antibodies, and vaccines, particularly in pediatric populations. Its pathogenesis remains incompletely understood, but proposed mechanisms include immune complex formation, altered drug metabolism, lymphocyte toxicity, and the development of anti-drug antibodies. Diagnosis is primarily clinical, although novel diagnostic tools are emerging. Management involves discontinuation of the offending agent and supportive care, such as antihistamines or nonsteroidal anti-inflammatory drugs (NSAIDs) in mild cases, and corticosteroids in more severe cases. Long-term management, especially in cases requiring potential re-exposure to the causative agent, remains challenging. Skin testing and graded oral challenges appear promising within a structured clinical framework. Increased awareness of SSLR is essential for timely recognition and appropriate care, and further research is needed to elucidate its mechanisms and inform evidence-based management strategies.

## 1. Introducing the Framework of Serum Sickness-Like Reaction

Serum sickness-like reaction (SSLR) is a rare, immune-mediated condition characterized by a benign systemic inflammatory response, primarily affecting the skin and joints, following exposure to certain medications, vaccines, or infectious agents [1,2,3,4,5,6]. The classical clinical triad of SSLR consists of fever, rash, and joint pain or inflammation [6,7,8,9,10]. These clinical symptoms closely resemble those of classical serum sickness (SS), a prototypical type III hypersensitivity reaction mediated by immune complex deposition following exposure to heterologous protein antigens. Although SSLR resembles the clinical presentation of SS, they differ in their causes, underlying mechanisms, and histopathological characteristics. SSLR is usually triggered by non-protein agents, such as antibiotics or viral antigens, and is not consistently linked to immune complex formation, low complement levels, or vasculitis, which are key features of actual SS [1,11,12,13,14,15]. Therefore, SSLR is often considered a clinical mimic of SS rather than a direct immunopathological equivalent [1,16,17,18]. As summarized in Table 1, SSLR and SS differ substantially across multiple domains, including causative agents, underlying immune mechanisms, clinical course, laboratory findings, and tissue pathology. Distinguishing between SS and SSLR mainly serves epidemiological, etiological, and pathophysiological purposes. In clinical practice, their differentiation is based on the causative agent, the levels of the complement, and the histopathology. Although the clinical presentation, diagnostic investigation, and treatment approach in SS and SSLR are similar, SS generally has a slightly worse prognosis, emphasizing the importance of identifying end-organ complications, such as nephropathy, during the patient’s course. Thus, patients with SS are more likely to receive aggressive treatment with corticosteroids or plasmapheresis. Notably, it remains unclear whether monoclonal antibodies cause SS or SSLR, which confuses many physicians; however, the management remains the same [4,12,13,19,20,21,22,23,24,25].

But what is more to be explored? SSLR presents diagnostic and therapeutic challenges due to its heterogeneous etiology and overlap with other disorders. Although generally benign and self-limiting, SSLR can cause considerable morbidity, frequently leading to unnecessary investigations or inappropriate treatments when unrecognized [18]. Currently, diagnosis is primarily clinical, as no standardized confirmatory tests exist [14,18,25,26,27,28]. Laboratory tests are useful mainly for excluding alternative conditions but have limited sensitivity and specificity [4,5,7,18,25,26,27,29,30]. Histopathologically, SSLR is classified within the urticaria-like dermatoses spectrum [5,6,31,32], although its precise pathological correlates remain poorly defined. Despite potentially alarming symptoms, SSLR usually follows a benign, self-resolving course [5,12,29,33,34,35]. Severe complications or fatalities are rare, although the condition may result in considerable morbidity, frequent misdiagnosis, and avoidable hospitalization [2,3,5,25,35,36,37]. Management is predominantly supportive, focusing on discontinuation of the offending agent and administration of antihistamines and non-steroidal anti-inflammatory drugs (NSAIDs) for mild cases, with corticosteroids reserved for severe presentations [2,4,6,7,29,38]. Plasmapheresis is considered only in refractory cases [1,34,39,40]. A major unresolved issue is determining the safety of re-exposure to the same or related agents, a question that remains controversial despite advances in skin testing and graded oral challenge protocols [5,41]. Thus, the aim of this review is to provide a comprehensive narrative synthesis of the current literature on SSLR, including etiology, incidence and epidemiology, pathophysiology, histopathology, clinical presentation, diagnostic approach, therapeutic strategies, and long-term management. By integrating recent findings with established knowledge, this review seeks to highlight diagnostic challenges, inform clinical decision-making, and identify areas requiring further research.

## 2. Etiological Spectrum of SSLR: Shifts from Drug-Induced to Infection-Associated Triggers

Table 2 summarizes the main categories of implicated agents and triggers reported in studies that extend beyond isolated case reports, including basic research, observational cohorts, and systematic reviews. As a result, the causal association for these agents is supported by stronger and more consistent evidence [4,6,11,17,20,42,43,44,45,46,47,48,49,50,51,52,53,54,55,56,57,58,59,60,61,62,63,64,65,66,67,68,69,70,71,72,73,74,75,76,77,78,79,80].

### 2.1. Β-Lactam Antibiotics

B-lactam antibiotics are more closely associated with SSLR than any other class of antibiotics [3,6,7,57,81,82]. More specifically, the most reported antibiotics related to SSLR are cefaclor and amoxicillin [6,7,17,36,57,64]. The estimated incidence of cefaclor-induced SSLR ranges from 0.024% to 0.5% [1,3,8,36,83,84]. In comparison, the incidence of amoxicillin-induced SSLR is approximately 0.0074% [25,36]. Older studies indicate that cefaclor has a higher incidence of SSLR than amoxicillin [1,36,64,73,84]. Interestingly, Vial et al. [36] described 27 cases of SSLR among 137 cefaclor-associated reactions reported to the French Network of Regional Pharmacovigilance Centers, with 23 cases occurring in children under five years of age. Their analysis suggested an incidence of 0.024–0.2% per course of cefaclor, highlighting both the predominance in young children and the generally benign outcomes despite occasional severe presentations. Similarly, Lowery et al. [84] reported four cases of SSLR during cefprozil therapy, two of which occurred in patients with a prior history of cephalosporin-related reactions, suggesting a possible role of individual susceptibility and prior sensitization. Although these studies reinforced the higher risk associated with certain cephalosporins, they also illustrated the limitations of available data, which were largely based on spontaneous reports and small series, making precise incidence estimates difficult. However, recent findings suggest that amoxicillin may now be the leading cause of pediatric SSLR, possibly due to the decreased use of cefaclor in modern clinical practice [7,11,20,37,46]. Interestingly, two or more exposures to cefaclor may increase the likelihood of developing SSLR, which suggests an initial sensitization to cefaclor after the first exposure [17,35,73,85]. However, this observation does not apply to amoxicillin-induced SSLR [73,85].

Minocycline and trimethoprim-sulfamethoxazole (TMP-SMZ) are also linked with SSLR [36,37,64,86]. The incidence of SSLR due to TMP-SMZ has been reported to be 0.089% [36,83]. The incidence of ampicillin-induced SSLR is approximately 0.5%, with a prevalence that is roughly twofold higher among children under 10 years of age [73].

The question of cross-reactivity among β-lactam antibiotics in the context of SSLR remains unresolved: while some authors suggest a potential class effect [9,23], others argue that cross-reactivity plays little or no role [9,23,36,47,52,84,87,88]. A similar controversy exists in the literature about the future use of other antibiotics, such as tetracyclines in patients with minocycline-induced SSLR [23].

### 2.2. Monoclonal Antibodies

The role of monoclonal antibodies remains debated. Some researchers argue that, due to their protein structure, these biologics more commonly induce classical SS rather than SSLR, whereas others report SSLR-like presentations with chimeric and even humanized antibodies [37,89,90]. The most common monoclonal antibodies associated with SSLR are chimeric (25% of Fab fragment is murine), followed by humanized (2–5% of Fab fragment is murine) and human (no murine component) [29,39,75,91]. This explains why the chimeric monoclonal antibodies infliximab (IFX) and rituximab (RTX) are the two most common biologics causing SSLR, while the humanized omalizumab is reported as the third most common monoclonal antibody associated with SSLR [37]. A plausible explanation is that the immune system is more likely to develop antibodies against the larger murine component in the chimeric monoclonal antibodies [39,68,91,92]. According to various studies, the incidence of IFX-induced SSLR has been reported to range from 0.3% to 9% [39,60,65,66,68,75,93]. For RTX, the estimated incidence of SSLR is between 2.44% and 8% for patients being treated for primary Sjögren syndrome, while it is around 1.67% for those treated for immune thrombocytopenic purpura [50,90,94]. In the case of omalizumab, the incidence of SSLR is estimated to be between 0.4% and 0.6%, but it can be as high as 25% in patients with mast cell activation disorders [39].

Various risk factors have been linked with SSLR due to monoclonal antibodies. Specific risk factors for IFX-induced SSLR include retreatment with IFX after a long interval of months/years [29,68]. Autoimmunity and hypergammaglobulinemia are potential risk factors of RTX-induced SSLR [39,95]. Patients with lymphoproliferative disorders might be less likely to develop immune reactions against RTX due to severe immunosuppression, but hypergammaglobulinemia may lead to the production of antibodies against antigenic epitopes of RTX, leading to SSLR [95]. Moreover, long-term use of corticosteroids may increase the risk of omalizumab-associated SSLR, while selective immunoglobulin A (IgA) deficiency has been proposed as a risk factor for dupilumab-induced SSLR [39,96].

Furthermore, a dose-dependent relationship may contribute to the development of SSLR in the context of monoclonal antibody therapy [97,98]. Positive serology for antibodies against the administered monoclonal antibodies has been proposed as a general risk factor for SSLR due to monoclonal antibodies [68,90,99,100,101,102]. Remarkably, exposure to rodents may predispose to the formation of human antichimeric antibodies (HACAs) and the development of infusion reactions after exposure to chimeric monoclonal antibodies [92]. However, this association remains controversial, as findings from the ACCENT I clinical trial did not demonstrate a statistically significant relationship between SSLR and the presence of anti-IFX antibodies [51]. Consequently, the co-administration of monoclonal antibodies with adjunctive immunosuppressive agents, such as corticosteroids, may reduce the formation of HACAs and thereby mitigate the risk of infusion reactions, including SSLR [50,51,68,92,101,103,104,105].

### 2.3. Vaccines

Lastly, the relationship between SSLR and vaccines remains controversial. Although cases have been described following rabies, influenza, HBV, tetanus, measles–mumps–rubella (MMR), pneumococcal, and more recently coronavirus disease 2019 (COVID-19) vaccines, causality is not always clear and may depend on host or immunologic factors [19,106]. Among vaccines, rabies and influenza appear most frequently implicated [1,3,7,12,19,22,34,57,74]. For example, the incidence of SSLR after administering the human diploid cell rabies vaccine (HDCRV) has been reported to be 2.06% [74]. Patients who exhibit a high IgG response (greater than 2.5 IU) and have a history of previous immunization against rabies, whether with HDCRV or hamster kidney cell rabies vaccine (HKCRV), are at an increased risk for SSLR [74]. Furthermore, Galeano et al. [107] reported a case of SSLR following rabies postexposure prophylaxis with human rabies immunoglobulin (HRIG) and multiple doses of human diploid cell rabies vaccine (HDCV), complicated by cholestatic hepatitis and biliary stenosis, underscoring that although rare, rabies prophylaxis can be associated with clinically significant systemic reactions.

For influenza vaccines, the reported incidence varies: one study found a 3% risk following the inactivated Government Pharmaceutical Organization-Mérieux Biological Products (GPO-MBP) formulation, whereas Apisarnthanarak et al. [61], observed no SSLR cases among 50 patients who received Fluarix, though the difference was not statistically significant [61]. A positive history of food or drug allergy predisposes to SSLR after exposure to the inactivated influenza vaccine [61]. In addition, SSLR secondary to pneumococcal vaccination usually occurs in pediatric patients [34]. Chung et al. [34] reported one of the first cases in an immunocompetent adult. Typically, SSLRs develop within one to two weeks of initial exposure, and should be considered in patients presenting with fever, rash, and arthritis when the timing of symptom onset is consistent with vaccination. However, evidence linking pneumococcal vaccines to SSLR remains limited to isolated case reports, which restricts generalizability and makes it difficult to establish causality. The fact that most cases occur in children may reflect higher vaccine utilization in this group, rather than a true age-related predisposition. Overall, while pneumococcal vaccine–induced SSLR appears biologically plausible, the strength of the evidence is weak, and reported cases should be interpreted with caution [34].

More recently, attention has turned to COVID-19 vaccines. Alzaidi et al. [19] reported a case of SSLR following the first dose of the AstraZeneca ChAdOx1 nCoV-19 vaccine in a previously healthy young adult. The patient’s symptoms resolved with supportive management, and on follow-up her inflammatory markers normalized within one month, at which point clinicians advised against a second dose of the same vaccine. Similarly, Chaijaras et al. [12] described a severe SSLR occurring four days after administration of an inactivated COVID-19 vaccine, which required a prolonged course of systemic corticosteroid therapy and precluded further vaccination. These cases add both adenoviral vector-based and inactivated COVID-19 vaccines to the expanding list of potential SSLR triggers. However, the strength of evidence remains limited, as the available data are restricted to single case reports, some of which may have been previously subsumed under broader categories of hypersensitivity reactions [108]. Recognition of SSLR in this context is clinically important, since it often prevents subsequent vaccination unless benefits clearly outweigh risks. Although a few successful desensitization attempts have been reported, no standardized approach currently exists [109].

### 2.4. Infectious Agents and Other Interactions

While medications have traditionally been blamed for SSLR, more recent data indicate that viral infections are becoming a primary cause, especially in children [5,41,86]. For instance, acute hepatitis B infection has been reported to lead to SSLR in up to 20% of cases [110]. Viral-drug or drug–drug interactions might explain why some patients do not experience recurrence upon re-exposure to these agents [5,18,20,37,46]. Respiratory viruses [46,110,111,112,113,114,115,116], Mycoplasma, and Streptococcus species are commonly implicated microbial triggers [5,13,14,19,46,110,111,112,113,114,115,116].

## 3. Epidemiologic Patterns, Risk Factors, and Drug Associations of SSLR

### 3.1. Incidence and Prevalence

Generally, the true incidence and prevalence of SSLR remain poorly defined [6,57,117]. A historical estimate from 1992 suggested an annual incidence of 4.7 per 100,000 children under 16 years of age [36]. Reported rates vary considerably according to the offending agent: bupropion is associated with extremely low estimates 0.002% [62], whereas cefaclor has been reported in up to 0.5% of exposed patients [69,73,118], and certain monoclonal antibodies in up to 8% [70]. Vaccines such as rabies and influenza have been linked to rates of 2–3% in some reports [61,74] (Table 3). Despite these figures, SSLR is likely underrecognized, as its clinical features overlap with more common conditions, specific diagnostic tests are lacking, and many cases are mild or self-limiting [75]. Consequently, SSLR should be regarded as uncommon in absolute terms, but plausibly underdiagnosed. Moreover, the apparent increase in published reports may reflect greater clinical awareness and changing prescribing patterns, such as reduced cefaclor use with a relative rise in amoxicillin-associated cases, rather than a proven rise in true population incidence.

### 3.2. Age-Related Patterns

The relationship between age and SSLR is noteworthy (Table 4). Cases occur more frequently in children, typically within one to two weeks of initiating antibiotic therapy for respiratory tract infections [4,7,35,36,37,57,64]. This higher prevalence may partly reflect the greater burden of infections and more frequent or prolonged antibiotic use in this group. However, developmental differences in drug metabolism, immune responsiveness, and pediatric formulations may also contribute [7,16]. Supporting this, a 10-year retrospective cohort study of 83 pediatric patients reported a mean age at presentation of 3.2 years, with amoxicillin implicated in over 80% cases. The mean latency to symptom onset was 8.5 days, and clinical manifestations included urticaria-like or erythema multiforme-like eruptions in most patients, joint inflammation in 60%, and pruritus, fever, or mucosal swelling in a substantial proportion. Incremental T-cell toxicity was demonstrated by the lymphocyte toxicity assay in most tested patients, and recovery tended to be faster in children receiving corticosteroids in addition to antihistamines/NSAIDs, although the difference did not reach statistical significance (6 vs. 8 days; *p* = 0.09) [7]. However, although some authors regard oral provocation testing as a safe diagnostic approach for assessing drug causality in SSLR [121], the potential recurrence of symptoms may be distressing and uncomfortable, particularly for young children [7].

More recently, a multicenter retrospective analysis of 171 pediatric cases highlighted diagnostic and therapeutic challenges, with SSLR considered in the initial differential diagnosis in only 29% of cases. Nearly half of affected children required hospitalization, and treatment strategies varied considerably, with over 50% receiving systemic corticosteroids alongside antihistamines and NSAIDs [45]. The decision to hospitalize children appears multifactorial, influenced by parental concern over impaired mobility and the need to exclude serious differential diagnoses such as septic arthritis or severe drug hypersensitivity with facial edema [45].

Moreover, corticosteroids are frequently prescribed for pediatric SSLR. However, evidence supporting their benefit remains inconclusive. Available studies show only a nonsignificant trend toward faster recovery with corticosteroids compared with antihistamines or NSAIDs, and no randomized controlled trials exist. Given the potential adverse effects even with short-term use, corticosteroid therapy should be carefully individualized in children. While prescribing patterns appear similar in adults, the risk–benefit balance may differ in pediatrics due to growth-related concerns and the typically self-limited course of SSLR [6].

Nevertheless, SSLR has been reported across all age groups, including the adult population [3,4,5,37,77]. Certain antibiotics demonstrate age-related patterns in their association with SSLR; for example, minocycline is predominantly implicated in adolescents and young adults, particularly those undergoing treatment for acne [77,122], whereas trimethoprim–sulfamethoxazole (TMP–SMZ) is most frequently reported in individuals with a median age of 31 years, and cephalexin in those with a median age of 53 years [36,64,77,122]. In contrast, monoclonal antibodies and antidepressants (e.g., bupropion, selective serotonin reuptake inhibitors) are more commonly associated with SSLR in adults, likely reflecting the greater utilization of these agents within this demographic [37,62]. Thus, while age patterns largely mirror drug utilization trends, pharmacokinetic and immunologic differences across the lifespan may also play a contributory role.

### 3.3. Sex and Genetic Factors

Sex is generally not considered a risk factor for severe SSLR [12,20,35,57,62,65,86]. However, the apparent absence of sex-related differences in pediatric cohorts may be influenced by study design, as most available studies recruit approximately equal proportions of male and female patients [20,57]. Moreover, some patients with SSLR have reported a family history of allergies to the same drug, but this genetic association has not been extensively studied [7,17,20,123]. Interestingly an in vitro study on cefaclor-induced SSLR suggests a possibility of maternal inheritance, as family studies demonstrated that five of six mothers who had never received cefaclor nonetheless exhibited a positive cytotoxic response, consistent with a maternal inheritance pattern [47]. Additionally, a history of atopy might be a risk factor, particularly for females [48,61,87].

### 3.4. Dose-Dependence and Host Factors

Many researchers believe that SSLR is a host-dependent, idiosyncratic reaction, but a dose-related relationship has been suggested for certain medications [47,48,49,62,97,98,124]. There have also been cases where SSLR developed after increasing the dosage of naproxen and omalizumab [97,124]. The risk of SSLR associated with penicillin and monoclonal antibodies may increase with higher doses [49,98].

## 4. Pathophysiology of SSRL

The pathogenesis of SSLR is not fully understood. While several theories have been proposed, only a few have strong support from experimental data. One theory suggests that infectious agents, particularly viruses, along with their interaction with certain medications, may explain a significant number of SSLR cases. This is supported by observations that SSLR typically does not recur during graded oral challenge tests or after subsequent treatments with the same medication [3,20,46,125,126].

Another theory proposes that hepatic biotransformation of administered non-protein drugs into immunogenic metabolites may underlie the development of SSLR [11,47,52]. In this model, the accumulation of lymphotoxic metabolites, coupled with an impaired capacity of lymphocytes to detoxify these compounds in genetically predisposed individuals, results in lymphocyte death and subsequent inflammation [11,16,19,123]. Τhis hypothesis has been supported by a pilot study in which Elzagallai et al. [11] demonstrated a significant, concentration-dependent increase in lymphocyte cytotoxicity in patients with suspected β-lactam–induced SSLR compared with healthy controls using the lymphocyte toxicity assay, suggesting a potential role for this test in diagnosis. Further clinical evidence is provided by Bakhshi et al. [123], who reported a case of a 4-year-old boy developing a severe SSLR with angioedema and systemic symptoms after re-exposure to amoxicillin only two months following an initial uneventful course, highlighting the risk of sensitization and accelerated reactions upon repeat exposure.

However, this mechanism has been most clearly implicated in cases of cefaclor-associated SSLR [47,52]. Interestingly, two experimental studies have proven that their metabolites mediate the toxic effects on lymphocytes in patients with cefaclor-associated SSLR [47,52], however they were based on small in vitro cohorts, and their clinical generalizability remains uncertain. In one study, lymphocytes from affected patients demonstrated a 100% greater rate of cell death compared with controls, and family testing suggested a maternal inheritance pattern of susceptibility [47]. In a follow-up study, patients with cefaclor-associated SSLR exhibited marked in vitro cytotoxicity to cefaclor metabolites (83.6 ± 42.2% cell kill above baseline), whereas loracarbef metabolites did not produce significant toxicity, a finding consistent with therapeutic rechallenge in three children who tolerated loracarbef without adverse effects [52].

The same hypothesis may apply to antibiotics such as penicillin G and tetracyclines [123]. A similar mechanism is well known to be responsible for the development of hypersensitivity reactions due to sulfonamides and aromatic anticonvulsants [84,125]. The recognition of a specific drug by a specific clone of T helper and memory cells is an essential immunologic mechanism [127]. Additionally, many authors support the hypothesis that small, non-protein drugs can act as haptens, binding to host proteins and initiating an inflammatory response [1,13,16].

Moreover, the formation of anti-drug antibodies (ADAs) has mainly been involved in the pathophysiology of SSLR due to monoclonal antibodies [50,68,95,102,104,105]. Notably, researchers have discovered two sites in the molecule of dupilumab that function as ADA binding sites [96]. However, there is conflicting evidence about the role of ADAs in SSLR secondary to monoclonal antibodies [51]. To be more precise, studies supporting ADA involvement often focus on highly immunogenic agents such as infliximab, whereas studies showing no association typically involve smaller cohorts or biologics with lower immunogenicity antibodies [50,68,95,102,104,105]. This inconsistency likely reflects heterogeneity in drug mechanisms rather than a uniform absence of ADA contribution [95]. Nevertheless, most case or case series reports are observational and involve a small number of patients, raising the possibility of reporting bias and highlighting the need for prospective studies with larger sample sizes and standardized immunologic assays. For instance, Finger and Scheinberg [95] reported that, during follow-up, patients tested negative for the development of human antichimeric antibodies when assessed with a commercial assay. In contrast, Grillo-Lopez et al. [128], observed antibody formation in only three patients treated with a chimeric monoclonal antibody, none of whom developed clinical features of SS. These findings suggest that the mere presence of ADAs may not be sufficient to precipitate SSLR and highlight the importance of host- and disease-related factors in modulating clinical outcomes.

There are also reports of ADAs in SSLR cases caused by β-lactams and HDCRV [25,74]. For example, Warrington et al. [74] reported SSLR following HDCRV, in which patients demonstrated IgE and IgG antibodies directed not against rabies antigen itself but against vaccine excipients such as fetal calf serum, suggesting that non-viral components may underlie the reaction. In contrast, Tatum et al. [25] described severe SSLR in adults receiving oral penicillins, but no specific antibody responses were identified, and diagnosis remained clinical. These findings underscore both the heterogeneity of immunologic pathways implicated in SSLR and the limitations of available evidence, which is largely restricted to case reports without standardized laboratory confirmation. In addition, these preformed antibodies can explain why the recurrence of SSLR is more severe and faster in some patients after repeated drug exposure [129].

Despite being a hypersensitivity reaction to viral and/or pharmacological agents, SSLR is not included in the Gell and Coombs classification. Older research has proposed that SSLR may represent either a type I or type III hypersensitivity reaction; however, the supporting evidence remains limited and conflicting. Although SSLR often exhibits clinical features resembling urticaria, it is not generally classified as a type I hypersensitivity reaction [84]. A case report with a combination of anaphylaxis and SSLR in a patient has highlighted that the possibility of a concomitant type I hypersensitivity reaction and SSLR should not be excluded [130]. The role of IgE remains debated, with many authors claiming that SSLR is not IgE-dependent, given the delayed onset after exposure [7,48,52,59,131]. However, isolated studies have detected specific IgE against β-lactam antibiotics and even components of the HDCRV in patients with SSLR [68,74].

Furthermore, histamine might be an inflammatory mediator of SSLR, explaining the presence of pruritus [5]. For example, bupropion has been hypothesized to enhance histamine release through a mechanism analogous to that of amphetamines [62]. If the theory of type III hypersensitivity reaction is true about the pathophysiology of SSLR, the IgE and the anaphylatoxins (derived from the complement activation) should be involved in the local release of biogenic amines such as histamine and serotonin by mastocytes [25,40,50,53,132,133,134,135]. Consequently, increased vascular permeability is observed contributing to the tissue deposition of complement, ICs (IgG/IgM), and inflammation in various sites [25,53]. The influx of neutrophils is also implicated in the pathogenesis of SSLR [89,134]. Evidence remains limited, but a case series of four patients receiving bupropion therapy reported leukocytosis with neutrophil predominance in one patient, suggesting a potential role for neutrophilic activation [134]. Conversely, a case report of a patient treated with alemtuzumab for multiple sclerosis described neutrophils as potential effectors of tissue damage in the context of SSLR, providing theoretical rather than experimental support for neutrophil involvement [89].

Moreover, the fact that infectious diseases like hepatitis B, hepatitis C, and infective endocarditis are causes of SSLR also implies that cryoglobulins and immune complexes (ICs) are involved in the pathogenesis of SSLR [3,110]. The normal serum levels of complement, the absence of anti-C1q antibodies, the absence of circulating ICs, the absence of vasculitis in skin biopsy, and the rarity of internal organ involvement in the majority of SSLR patients are considered essential for the differentiation from SS which is a classic example of type III hypersensitivity reaction [10,17,47,87,136,137].

Other possible mechanisms involved in the pathogenesis of SSLR are briefly reported:

- Elevated prostaglandins in omalizumab-induced SSLR production [138];
- Coagulopathy with hemolysis and saturation of the reticuloendothelial system by ICs or drug-protein complexes causing SSLR [139,140];
- Graft-versus-host-like reactions [36];
- Increased intestinal permeability in cefaclor-associated SSLR [48];
- Certain signaling pathways in SSLR due to anti-TNF agents used for the treatment of inflammatory bowel disease [65].

In summary, current evidence most strongly supports a metabolite-mediated pathway and immune complex formation with complement activation and neutrophil recruitment, while the roles of anti-drug antibodies, IgE, and other proposed mechanisms remain largely theoretical or based on limited case reports.

## 5. Histopathology of SSLR

SSLR is primarily a clinical diagnosis; therefore, skin biopsy is warranted only in equivocal cases to exclude other morphologically similar conditions [7,32,141,142]. Histopathologically, SSLR is classified within the spectrum of urticarial disorders [6,31,32,142,143,144]. The predominance of neutrophils often highlights its similarity to neutrophilic urticaria and neutrophilic urticarial dermatosis [8,31,34,143]. In some cases, lymphocytes, predominantly T cells or eosinophils constitute the dominant inflammatory cell population [96,145]. Typical histopathological features include a perivascular and interstitial infiltrate of mixed inflammatory cells, including neutrophils, lymphocytes, and eosinophils, with dermal edema and ectatic blood vessels [7,31,32,96,142,143,146]. The location of the interstitial infiltrate varies; superficial, mid-dermal, deep-dermal, and peri-adnexal positions have been reported separately or in combination [7,8,31,34,145,146].

The intravascular location of inflammatory cells has also been reported [96,142]. The epidermis may develop edema (spongiosis) [31,143,145]. The absence of vasculitis, fibrinoid vessel wall necrosis, deposition of ICs, and complement is characteristic of SSLR [12,14,22,23,142]. However, atypical cases with characteristics similar to SS, such as leukocytoclastic vasculitis and positive direct immunofluorescence (DIF) for complement or ICs have been reported in the literature [3,14,34,74,146,147]. These cases provide weak evidence for the theory of type III hypersensitivity reaction in the pathogenesis of SSLR. The differential diagnosis for the pathologist includes neutrophilic urticaria, neutrophilic urticarial dermatosis (NUD), interstitial granulomatous dermatitis, non-bullous neutrophilic lupus erythematosus, adult Still ‘s disease, cryopyrin-associated periodic syndrome (CAPS), Schnitzler’s syndrome, urticarial vasculitis (UV), urticarial hypersensitivity-type drug reaction, and SS [31]. The main difference between SSLR and NUD is that SSLR represents a transient drug-induced reaction, but NUD is a dermatologic complication of rheumatic diseases [31].

## 6. Clinical Presentation

A comprehensive delineation of the characteristic symptoms is presented in this section to serve as the primary reference for subsequent discussions on differential diagnosis and long-term management. The clinical presentation of SSLR is highly variable in both onset and duration. Symptoms typically appear within 1–2 weeks after exposure to the causative agent and often resolve within 1–2 weeks once the drug is withdrawn [4,35,57,61,62,148]. Patients may experience an accelerated onset upon re-exposure, particularly if previously sensitized, though this seems more characteristic of recurrent SS [41], than SSLR [4,6,28,106,138]. Clinically, SSLR can emerge as early as 24–36 h after drug administration or may be delayed for up to 4–6 weeks [10,17,61,138]. The duration of symptoms is equally diverse, ranging from short-lived episodes of 24–48 h to protracted courses lasting weeks or even several months, with rare reports extending up to 4 months [2,17,25].

**Figure 1:**
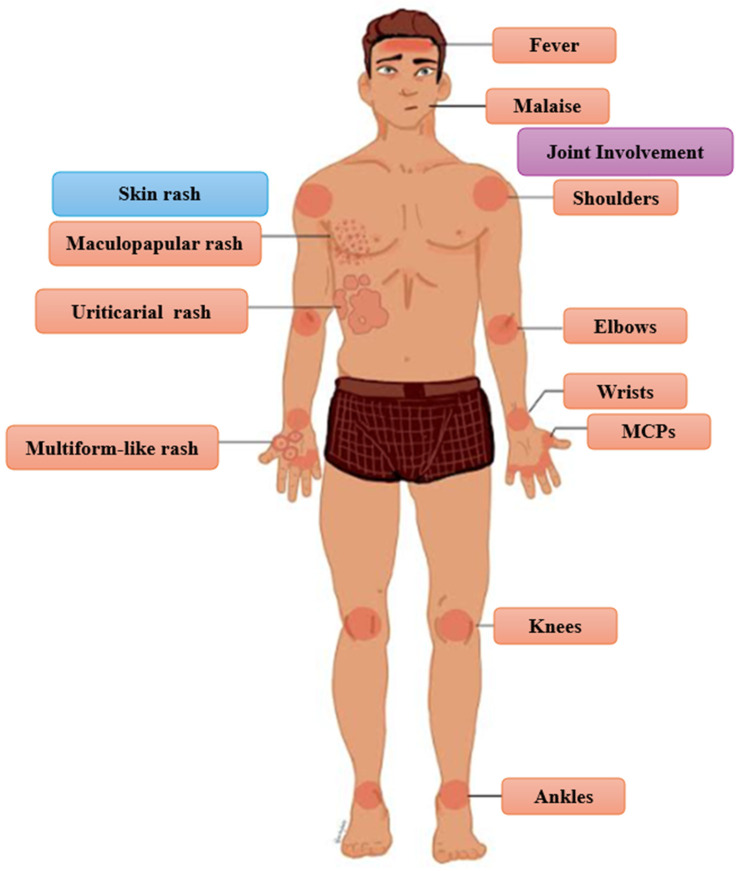
Classic Triad and Other Common Clinical Manifestations of Erythema multiforme-like rash in SSLR. MCPs: metacarpophalangeal joints.

Since SSLR is a clinical diagnosis, the physician should be well-informed about the symptoms, the signs, and their frequency [27,47]. The classic triad of clinical presentation includes rash, joint involvement, and fever, which is present in about half of patients with SSLR [91] (Figure 1). It must be noted that the classic triad was noted only in 28% of the patients with SSLR in a pediatric emergency department [4]. The frequency of skin eruption, fever, and joint involvement varies depending on the causative agent, the age population, and the definition of the study [4,6,7,13,20,54,57,58,77] (Table 5).

Although malaise is a non-specific symptom, it has been reported to be a universal symptom in patients with SSLR [7]. According to a recent systematic review, rash is present in all patients, fever in 35.1% of children and 72.3% of adults, and joint involvement in 88.2% of children and 84.1% of adults [6]. Other common symptoms (and their frequencies) are facial edema/angioedema (6.8–31%), gastrointestinal disturbance (67%), abdominal pain (6.4–21%), diarrhea (12.7%), and nausea (5.3%) [7,8,13,25,33,54,57,62].

Cutaneous manifestations of SSLR are heterogeneous and lack disease-specific characteristics. The morphology of the rash can be urticarial (16.7–41.8%), erythema multiforme-like (11.8–38.5%), and maculopapular (26.5%) [4,7,33,54]. Although most of the studies favor urticarial morphology, an epidemiologic study recently revealed that erythema multiforme-like rash is slightly more common than urticaria in patients with SSLR [7,20,57,77]. Pruritus is common and is reported in 34% of patients [7]. The occurrence of dermatographism remains a subject of debate, but it has been documented in some cases [5,22,32,38,126,149]. Additionally, cases of persistent hyperpigmentation and purpuric lesions following minimal trauma have been documented [14,137]. The joint involvement in patients with SSLR presents either as arthritis (intra-articular inflammation) or arthralgia due to edema of the overlying skin or periarticular inflammation [5,7,85,135]. The typical pattern of the affected joints follows the rules of symmetry and hand-foot involvement [3,7,20,62,85]. SSLR tends to affect the metacarpophalangeal joints (MCPs), and the large joints (wrists, elbows, shoulders, knees, ankles) [13,25,29,52,57]. Atypical presentations, including asymmetric arthritis or monoarticular involvement, have also been documented [134,150]. In pediatric patients, refusal to ambulate is a frequently reported clinical feature [2].

**Figure 2:**
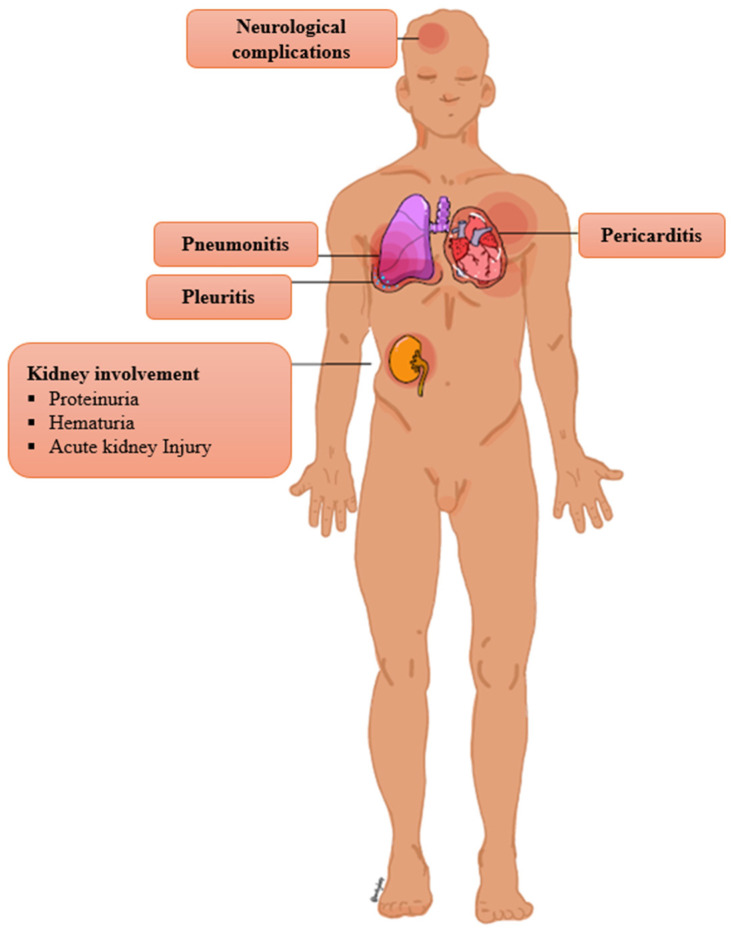
Rare complication of SSLR.

Moreover, involvement of lymph nodes and internal organs is a rare clinical manifestation of SSLR (Figure 2). Lymphadenopathy is generally regarded as an uncommon manifestation of SSLR, with reported frequencies ranging from 0.2% to 20% [8,13,17,27,28,30,57,122,150]. However, some authors contend that lymphadenopathy represents a common manifestation of SSLR [2,3,10,29,54,135]. Reported cases have described involvement of various anatomical regions, including the cervical, axillary, and inguinal lymph nodes, typically presenting as tender nodal enlargement [12,97,137,143,151,152,153]. Acute kidney injury is an uncommon presentation [13]. A variety of neurologic complications are considered rare, although subclinical neuropathy might be more common [3,13,25,147,154].

Dyspnea and chest pain are infrequent symptoms, but they should raise suspicion of pneumonitis, pleuritis, and pericarditis [29,63,122,155,156,157,158]. A study about pediatric SSLR estimates that 13.8% of patients present with pneumonitis, 3.4% with pleuritis, and 3.4% with pericarditis [13]. Severe eosinophilic pneumonia has been reported in the context of IFX-induced SSLR [68]. Hypotension is a rare finding; if present, it mimics anaphylaxis [106,159]. A case of compartment syndrome due to massive acral edema has been described [160]. Splenomegaly and hepatomegaly are uncommon signs [2,13,29,158,161]. A case of esophageal discomfort in a patient with IFX-induced SSLR raises suspicion of possible esophagitis in the context of SSLR [68]. SSLR has been reported to trigger preterm labor and diabetic ketoacidosis in pregnant and diabetic patients, respectively [40,162].

## 7. Differential Diagnosis of SSLR

The differential diagnosis of SSLR is broad and varies depending on the clinical presentation and the patient’s age (Table 6). Of course, infectious diseases should be considered depending on the clinical presentation, such as syphilis, viral exanthem, and streptococcal complications [18]. The most important alternative diagnoses are urticaria, urticarial vasculitis (UV), urticaria multiforme (UM), erythema multiforme (EM), drug reaction with eosinophilia and systemic symptoms (DRESS), Stevens-Johnson syndrome/toxic epidermal necrosis (SJS/TEN), exanthematous drug eruption, Kawasaki disease, and systemic juvenile arthritis (SoJIA). Urticaria causes pruritic skin lesions with central clearing lasting less than 24 h without systemic symptoms such as fever or articular involvement [1,9,13,18,33].

Differentiating SSLR from infectious causes of febrile rash can be challenging because of overlapping systemic symptoms and rash appearance. A thorough evaluation of the patient’s history (especially recent drug exposure) and rash is essential [18]. Secondary syphilis should be considered in sexually active patients, particularly a few weeks after a painless, indurated ulcer appears on the genital area; it typically causes an erythematous maculopapular rash on the trunk and extremities, along with fever, lymphadenopathy, and mucosal involvement, such as condyloma lata. However, SSLR usually does not include palmoplantar rash, mucosal lesions, lymphadenopathy, or a recent chancre [163]. Meningococcemia, whether with or without meningitis (Neisseria meningitidis infection), is a life-threatening illness presenting with a diffuse petechial, non-blanching rash, coagulopathy, and adrenal insufficiency. In contrast, SSLR involves a blanchable rash without petechial or purpuric features or signs of sepsis [163]. Rocky Mountain Spotted Fever (RMSF), caused by Rickettsia rickettsii, presents with fever, headache, and a blanching maculopapular rash beginning on the wrists and ankles, spreading to the trunk, palms, and soles, and can lead to serious complications like coagulopathy. Unlike RMSF, SSLR does not have this migratory rash pattern or severe complications [163]. Erythema marginatum consists of centripetally expanding erythematous, non-pruritic lesions with central clearing and a well-defined border. Although erythema marginatum is linked to fever and joint involvement in acute rheumatic fever (ARF), SSLR lacks this typical morphology and other ARF features such as migratory arthritis, carditis, chorea, subcutaneous nodules, and chorea [18,164,165,166]. Scarlet fever appears as a diffuse, blanching rash spreading from the trunk outward in the context of streptococcal pharyngitis. It is associated with Pastia lines, strawberry tongue, and circumoral pallor, which are absent in SSLR [18,164]. Rubella and measles present with a maculopapular rash that spreads downward and systemic signs of inflammation, mainly in unvaccinated children. Rubella is also associated with arthritis and low-grade fever, whereas measles features high fever and malaise. SSLR, however, does not show postauricular or suboccipital lymphadenopathy, viral prodrome, or Koplik spots [164,166]. Chickenpox (primary varicella-zoster virus infection) mainly affects unvaccinated children, manifesting as multiple pruritic vesicles with various morphologies that spread centrifugally in combination with systemic inflammation [167]. Still, SSLR lacks vesicles and presents with different types of rash (urticaria, erythema multiforme, and maculopapular). Hand, foot, and mouth disease, caused by coxsackievirus, involves painful ulcers in the posterior pharynx and a maculopapular or vesicular rash on the extremities [164], but SSLR does not cause mucositis or vesicles. Erythema infectiosum (fifth disease), caused by parvovirus, can show high-grade fever, joint involvement, and facial erythema (slapped-cheek rash) followed by a reticular rash on the trunk and extremities [166], a pattern not typically seen in SSLR.

Urticaria causes pruritic skin lesions with central clearing lasting less than 24 h; it typically lacks systemic symptoms, such as fever or articular involvement, in contrast to SSLR [1,9,13,18,33]. UV typically presents in young female patients as a long-standing disease with histopathologic evidence of leukocytoclastic vasculitis. Ιt is associated with hypocomplementemia, anti-C1q antibodies, internal organ involvement, and an underlying autoimmune or malignant disease, which are atypical features of SSLR [12,14,31,33,38]. In clinical practice, however, UV is often considered in the differential diagnosis of SSLR because both may present with urticarial rash, fever, and arthralgia. Key distinctions include the persistence of UV lesions for >24 h with residual purpura, systemic involvement, and hypocomplementemia, whereas SSLR is usually self-limited, complement levels are normal, and histology shows only nonspecific perivascular inflammation. Because of this overlap, SSLR may initially be misclassified as UV, making careful attention to rash duration, complement testing, and systemic features essential for accurate diagnosis [33].

In contrast, UM, also known as acute annular erythema, appears 1 to 3 days after a viral illness. It is marked by ecchymotic urticarial lesions that are itchy and exhibit dermatographism. The absence of systemic involvement, the excellent response to antihistamines, and the short latency period help differentiate UM from SSLR. [14,22,38,141,168].

EM is characterized by targetoid lesions with central purpura or blistering, often located on the palms and soles, and may include oral lesions. It is predominantly associated with herpes simplex virus (HSV) infections, but some cases occur after exposure to antibiotics and antiepileptics [169,170]. Although a significant portion of patients with SSLR develop EM-like rash, the lack of systemic symptoms and the close relationship with HSV favor the diagnosis of EM [9,17,22,38,87,168,169,170]. Notably, Nnomadim et al. [141] reported a 13-month-old girl with an atypical reaction to amoxicillin who was initially misdiagnosed with EM and later Henoch-Schonlein Purpura, until the recurrence of hives clarified the diagnosis as SSLR.

Another alternative diagnosis is DRESS, characterized by febrile rash, prominent lymphadenopathy, facial edema, eosinophilia, and internal organ involvement (e.g., hepatitis) in the absence of joint manifestations, typically arising 2–6 weeks after exposure to the causative agent, most often anticonvulsants or sulfonamides. Although the dermatological manifestations and the latency period of SSLR and DRESS are similar, DRESS requires the presence of additional diagnostic criteria. In previously sensitized individuals, symptom onset may occur more rapidly upon re-exposure, in some cases within 24 h [8,18,23,88]. SJS/TEN often mimics SSLR due to the development of targetoid lesions with centrifugal progression. However, SJS/TEN is associated with mucosal involvement, epidermal detachment (positive Nikolsky sign), and a prodromal phase, often characterized by upper respiratory tract symptoms resembling a viral illness, which are absent in SSLR [8,23,88,133,135,171].

Patients with exanthematous drug eruption present with a maculopapular rash spreading from the trunk to the extremities and may have mild fever without other signs of systemic disease [9,141]. Kawasaki disease is typically diagnosed in children (peak incidence in the second year of life who present with high-grade fever, diffuse skin rash in palmar-plantar distribution with late desquamation, mucosal involvement (bilateral conjunctivitis, cracked lips, and strawberry tongue), and cervical lymphadenopathy for at least five days. Typical features of Kawasaki disease include mucositis, lymphadenopathy, and palmoplantar rash, which are unlikely to occur in SSLR [2,3,81]. SoJIA requires the presence of arthritis (for at least 6 weeks), fever (for at least 2 weeks), rash, and signs of systemic disease such as lymphadenopathy, hepatosplenomegaly, serositis, or macrophage activation syndrome (MAS) in patients younger than 16 years old; fever spikes accompanied by transient worsening of arthritis and rash are characteristic [3,14,172]. Similarly, adult Still ‘s disease is differentiated by SSLR in older patients from the episodic nature of salmon-like rash, lymphadenopathy, internal organ involvement, and life-threatening complications [3,18,33,173,174].

The differential diagnosis of SSLR is further complicated by overlapping features with other systemic hypersensitivity reactions and inflammatory syndromes. Anaphylaxis, in particular, can mimic sepsis due to its systemic manifestations, including fever, leukocytosis, hypotension, and respiratory distress. Multiple inflammatory markers, including white blood cells (WBCs), C-reactive protein (CRP), procalcitonin (PCT), pancreatic stone protein (PSP), neutrophil extracellular trap (NETs), and interleukin-6 (IL-6), are typically linked with acute infection, sepsis, or septic shock as they are useful in diagnosis, prognosis, and monitoring [175,176,177,178,179,180]. However, the value of inflammatory markers should be interpreted in the appropriate clinical context because non-specific elevation is occasionally reported in hypersensitivity reactions. Mirijello et al. [181] described a patient with amoxicillin-clavulanate–induced anaphylaxis whose elevated PCT levels, leukocytosis, and chest radiographic abnormalities initially suggested septic shock, while Kim et al. [182] reported risedronate-induced anaphylaxis in a 74-year-old woman with marked CRP and PCT elevations, again masquerading as sepsis until drug rechallenge clarified the diagnosis. In agreement, Mann et al. [183] presented a patient with trimethoprim–sulfamethoxazole–induced anaphylaxis and a PCT concentration of 29 ng/mL, levels traditionally regarded as almost exclusively consistent with septic shock, yet cultures remained negative, and the patient improved with anaphylaxis management. While Bánvölgyi et al. [184], in a recent published article, reported two additional cases of TMP-SMZ-induced anaphylaxis initially misdiagnosed as sepsis due to high PCT concentrations, reinforcing that elevated PCT can occur in hypersensitivity reactions. It is therefore clear that PCT elevations, even at very high levels, lack absolute specificity for bacterial sepsis and may delay recognition of alternative diagnoses. For SSLR where no pathognomonic biomarkers exist and clinical overlap with sepsis, urticaria, and anaphylaxis is substantial these findings are particularly relevant. Diagnostic accuracy requires integration of drug history, temporal patterns, and biomarker trends over time, rather than reliance on isolated laboratory values, in order to avoid misdiagnosis and inappropriate management [183,185,186].

## 8. Diagnosis

The role of diagnostic testing in identifying SSLR remains unclear, as the diagnosis is primarily determined through clinical evaluation. Clinicians should suspect SSLR in patients with recent drug, vaccine, or infection exposure, typically within one to two weeks, who present with at least two of the following: fever, rash, and joint involvement, often accompanied by elevated inflammatory markers such as CRP, erythrocyte sedimentation rate (ESR), and white blood cells [6,7,9,13,23,33,59,88]. In pediatric patients who present with a benign general appearance and a high clinical suspicion of SSLR, laboratory investigations are generally deemed unnecessary [4,14]. SSLR is a diagnosis of exclusion; therefore, laboratory investigations are primarily utilized to rule out infectious and immunologic disorders, guided by the patient’s clinical presentation [18]. It is widely accepted that cell blood count (CBC), inflammatory markers, complement, serology, liver function tests (LFTs), and urine analysis are basic diagnostic trials that often reveal abnormalities, but their diagnostic value remains poor [5,25].

CBC may be positive for leukocytosis, neutrophilia, lymphopenia, eosinophilia, and thrombocytosis [23,57]. The sensitivity for CBC abnormalities for pediatric SSLR is thrombocytosis (50%), neutrophilia (32%), leukocytosis (22%), and lymphopenia (22%) [7]. Anemia, leukopenia, neutropenia, and thrombocytopenia have also been reported [19,27,28,30,61,151,156]. Other studies show 34.5% and 46% sensitivity towards leukocytosis [13,56]. The peripheral blood smear may reveal atypical lymphocytes and rarely plasma cells (reactive plasmacytosis) without bone marrow abnormalities [29,34,147,160,187].

Inflammatory markers such as ferritin, lactate dehydrogenase (LDH), ESR, and CRP are often elevated [12,14,19,34,81]. The reported diagnostic sensitivity of CRP is approximately 50%% [7], whereas ESR demonstrates a broader range, with sensitivities of 58.6% and 76% in different cohorts [13,56]. In addition, in contrast to classical SS, complement levels in SSLR are typically within the normal range [1,8,13,23,117]. Several studies support this distinction: for example, Yorulmaz et al. [13] did not analyze complement in detail because values remained normal, while Brucurelli et al. [188] described a cefazolin-induced SSLR case with preserved complement levels. Taking together, the current evidence suggests that complement testing has limited sensitivity or specificity for SSLR and should primarily be interpreted as a tool to differentiate SSLR from true SS rather than as a standalone diagnostic marker.

However, many exceptions question this rule [27,61,130,133,137,139]. Interestingly, hypocomplementemia was present in 83.3% of the patients involved in a study [7]. Serial measurements of the complement have been proposed to detect any fall from the baseline irrespective of the normal range of values [25,152]. Serology for anti-C1q antibodies is typically negative [12]. Serology for ICs is typically negative, but there are some exceptions [23,25,34,139,141,188]. For example, antinuclear antibodies (ANA) and anti–cyclic citrullinated peptide antibodies (anti-CCP) positivity have been reported, but further research is necessary [3,19,96,136,153]. Screening for HACAs has been proposed for SSLR secondary to monoclonal antibodies [68,100,102]. A comparable assessment for antibodies directed against streptokinase has likewise been reported [63].

Hepatic and renal involvement in SSLR is uncommon, and when present, it more frequently manifests as asymptomatic laboratory abnormalities rather than overt clinical signs. Liver function testing occasionally demonstrate elevated transaminase levels [19,29,62,89,99,189]. Urine analysis is sometimes positive for mild proteinuria, hematuria, or both [3,8,61,62,122,134]. Dysmorphic red blood cells have been observed, suggesting glomerulonephritis, although this is controversial [129].

Hematologic abnormalities in SSLR are uncommon. SSLR is a rare cause of transient M-spike and hypergammaglobulinemia [147,152,187]. In these cases, it is advised that plasma cell disorders should be excluded with further testing [187]. Abnormalities in the coagulation parameters have been reported [62,139,140]. Laboratory measurements consistent with hemolysis (decreased free haptoglobin, increased haptoglobin-hemoglobin complexes) and activation of the reticuloendothelial system (low fibronectin) were present in a case [139,140].

Imaging is rarely necessary when there is clinical suspicion of SSLR. Electrocardiogram, echocardiography, and chest X-rays are useful for patients presenting with chest pain or dyspnea [155,156,157,158]. Abdominal ultrasound revealed aseptic cholecystitis in a patient with abdominal pain and SSLR [99]. Imaging for acral edema with ultrasound reveals a subcutaneous anechoic fluid collection [123,129]. Imaging for arthritis with computed tomography (CT) shows edema and synovial enhancement [34]. Magnetic resonance imaging (MRI) revealed extensor tenosynovitis complicated by soft tissue necrosis in a case of omalizumab-induced SSLR [103]. Arthrocentesis was performed in some cases for the exclusion of septic arthritis; the results were indicative of inflammatory arthritis [26,34,153].

Special immunologic tests have been developed in older studies to confirm the clinical suspicion of SSLR. The radioallergosorbent test (RAST) has been proposed in the past, but a recent study indicated a lack of sensitivity, which contradicts the theory of type I hypersensitivity reaction [7,55]. The mast cell degranulation test (MCDT) and the migration inhibition factor (MIF), which estimate the IgE-mediated immune response and the cellular immunity, respectively, have been used successfully in a small series of patients with SSLR [136].

Increased intestinal absorption was observed in patients with cefaclor-induced SSLR 1–2 weeks after the initial dose of cefaclor [48]. The lymphocyte stimulation/transformation test (LST/LTT) has been used in older studies; the results revealed that the lymphocytes from patients with SSLR multiply in the presence of the responsible medication [49,127,131]. The results are reproducible months or years after the episode of drug reaction [49,131]. Recently, the lymphocyte toxicity assay (LTA) has been developed to confirm clinical suspicion of SSLR [7,11,52,84,189]. The LTA test is based on the concept that T-cells are vulnerable to the metabolites of cefaclor. Hence, the in vitro exposure to the metabolites leads to measurable lymphocyte death. The sensitivity and specificity of LTA for type IV drug-induced hypersensitivity reactions have been reported to be 80–99% and 75–89%, respectively, while its sensitivity in SSLR is over 90% in small series [7,11,52]. LTA is also considered a highly specific test because it lacks cross-reactivity with similar drugs; lymphotoxicity was not observed in cases of cefaclor-induced SSLR after exposure to cephalexin or loracarbef [47,52]. Its diagnostic accuracy makes it an ideal test for diagnosing SSLR and identifying the triggering agent in a non-invasive way, especially in equivocal cases. Unfortunately, this test is not currently available in routine clinical practice [7].

**Figure 3:**
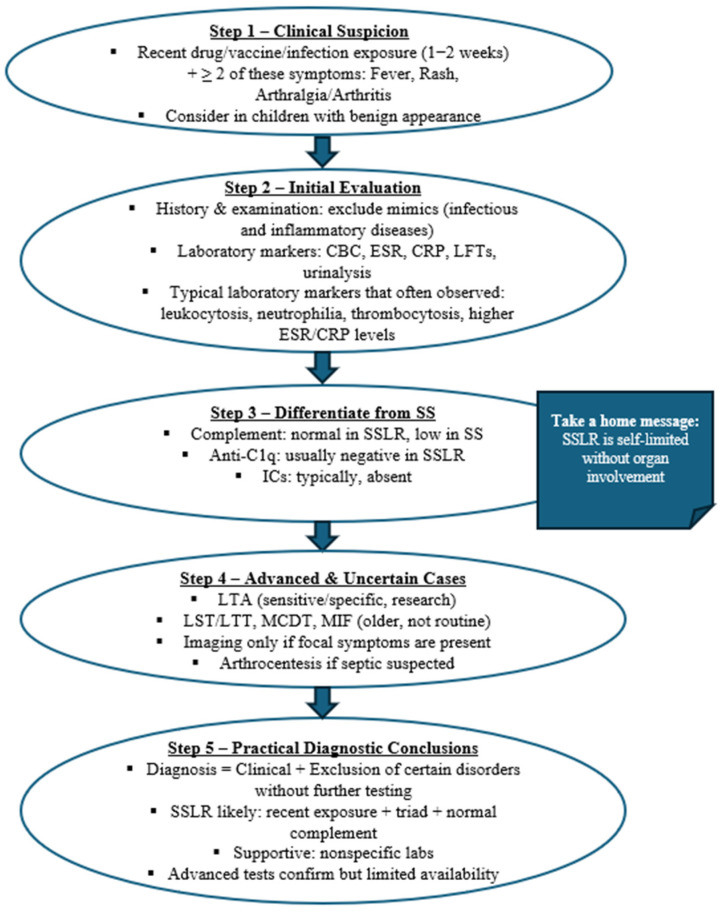
Proposed diagnostic algorithm for SSLR.

Taken together, SSLR should be approached as a clinical diagnosis supported by exclusion of mimicking conditions. The presence of the characteristic triad with recent exposure, preservation of complement levels, and absence of systemic organ involvement strongly favor SSLR, while advanced immunologic tests may support diagnosis in uncertain cases. Figure 3 illustrates a step-by-step diagnostic algorithm for SSLR, emphasizing recent exposure history, characteristic clinical features, and the exclusion of mimicking conditions. Supportive laboratory findings may aid recognition, while advanced tests are reserved for diagnostically uncertain cases.

## 9. Treatment

**Figure 4:**
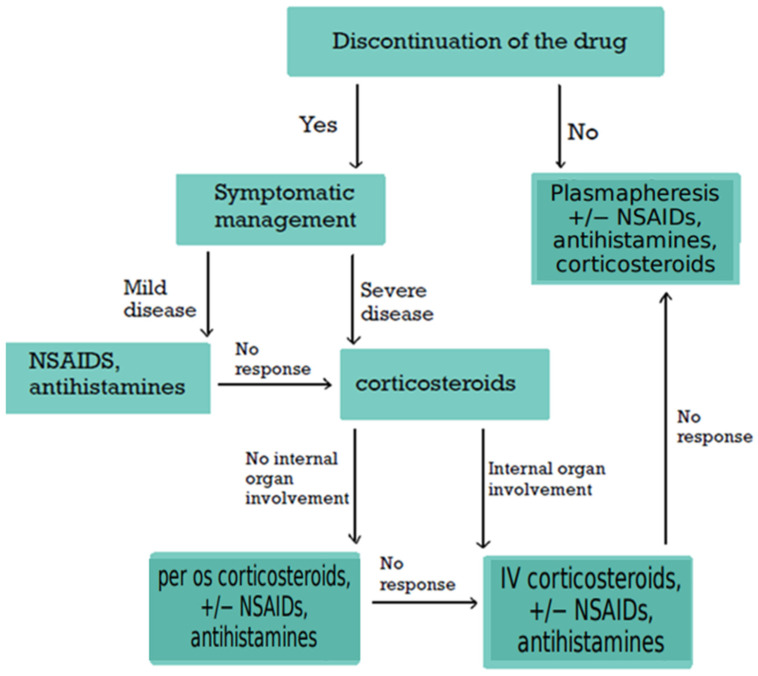
Proposed Treatment Algorithm for SSLR. Based on published case reports and expert opinion, this algorithm outlines the suggested approach to managing SSLR. Discontinuation of the causative agent and implementation of supportive measures remain the cornerstone of therapy. Further evidence is required to develop standardized, evidence-based guidelines. SSLR: Serum Sickness-like Reaction; NSAIDs: non-steroidal anti-inflammatory drugs.

SSLR is generally a benign and self-limiting condition, and management primarily focuses on removal of the underlying cause and provision of symptomatic relief (Figure 4). The available evidence remains limited owing to the absence of randomized controlled trials and formal guidelines [80,106]. The first step in treatment is discontinuation of the suspected causative agent. In cases where the responsible medication cannot be definitively identified, discontinuation of all concurrent medications has been attempted in a case report [106]. In rare instances where continuation of the causative agent is deemed essential, concomitant administration of plasmapheresis and/or corticosteroids or antihistamines has been proposed based on expert opinion from relevant cases [1,13,26,34,40,53].

It is important to note that SSLR is a self-limiting condition that typically resolves within a few days and only rarely persists for weeks or months [18,28,30,136]. In fact, spontaneous resolution was reported in an original article for the majority of 42 children with cefaclor-induced SSLR within one week after stopping cefaclor [48]. A typical course for the treatment of SSLR from diagnosis to resolution lasts approximately one week in various clinical studies [6,7,61,73]. NSAIDs are useful for the management of fever and arthralgia, and H1 antagonists are commonly prescribed for pruritus [1,2,4,7,28,34]. The wide use of NSAIDs and antihistamines is observed in multiple clinical studies [4,6,7,11,45,57,58,61,70,73,74]. According to a recent systematic review, children were more likely to receive non-steroidal agents relative to adults [6]. Sometimes, H1 antagonists are used in combination with H2 antagonists for additional relief, and doses of H1 antagonists up to four times the usual amount have been administered [30,123,150,188]. Other clinicians have used a combination of two H1 antagonists [57]. Additional management of the symptoms and complications is vital, e.g., topical corticosteroids, topical calamine, antiemetics, and fluids [9,17,23,35].

Lack of response to NSAIDs/antihistamines or severe symptoms are indications for treatment with corticosteroids [2,4,9,28,29,30,34]. Per os, intravenous (IV), or both routes of administration have been reported. Multiple clinical studies have demonstrated the role of corticosteroids in managing SSLR alone or in combination with non-steroidal agents [4,6,7,11,13,21,45,48,57,58,61,70,71,73,74]. A recent systematic review revealed lack of statistically significant difference regarding the administration of corticosteroids between children and adults with SSLR [6]. Interestingly, most cases of SSLR secondary to monoclonal antibodies reported in these clinical studies are treated with corticosteroids [58,70,71]. Treatment with corticosteroids was associated with a clinically significant difference in the duration of the disease; the difference was not statistically significant, perhaps due to the small number of patients in this study [7,123]. Although there is a lack of clinical data to compare the efficacy of different routes, IV corticosteroids followed by per os corticosteroids were preferred in three cases with SSLR and internal organ involvement (compartment syndrome, pneumonitis, or pericarditis) [156,157,160]. This is also a reasonable choice for those who cannot receive oral medications or have failed a trial of oral glucocorticoids as shown by a challenging case [122]. Discontinuation of glucocorticoids is done with a careful taper; rebound symptoms have been observed in published cases after rapid withdrawal [12,25,89]. Lack of response to corticosteroids is an indication of plasmapheresis; thus, this invasive option is considered last-line treatment option given the lack of data from clinical studies to support its efficacy [39,40].

## 10. Long-Term Management

**Figure 5:**
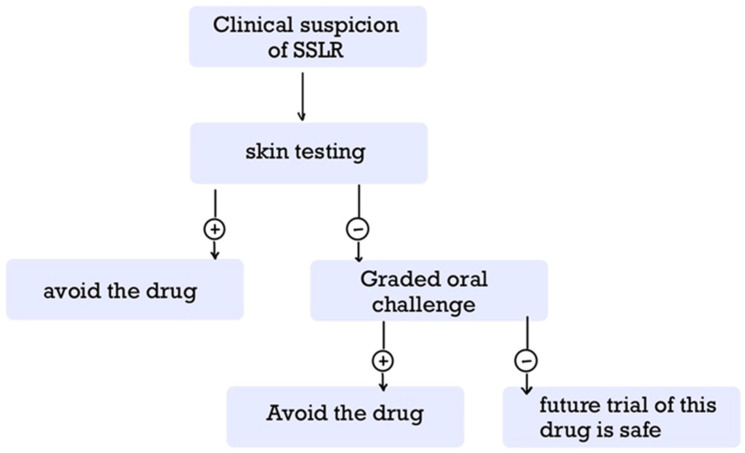
Proposed Algorithm for Long-Term Management of SSLR. This algorithm outlines a suggested approach to long-term management, incorporating skin testing and graded oral challenge to guide decisions regarding re-administration of the causative agent or a structurally related drug. SSLR: Serum Sickness-like Reaction.

Future management of patients with SSLR should consider their suitability for subsequent treatment with the same or a structurally related drug. This issue remains controversial. Re-exposure to the causative agent may precipitate a recurrence of SSLR or related disorders, such as urticaria or urticaria multiforme, and the clinical presentation may occur more rapidly and with greater severity than during the initial episode [81,106,129,131,144,151,189,190]. On the other hand, many patients have been able to tolerate subsequent courses of the same medication or have experienced only mild symptoms upon re-exposure [5,17,20,103]. Notably, patients with a positive history of drug allergy to antibiotics, e.g., β-lactams, are more likely to receive fewer effective antibiotics, leading to worse outcomes [5,20,123]. Recently, an algorithm including skin testing and graded oral challenges (GOCs) has been proposed to determine the patient‘s susceptibility profile [7,20,83,86] (Figure 5). It is recommended to apply this algorithm to all patients as soon as possible after the resolution of SSLR to minimize the use of allergic labels and the restriction of broad-spectrum antibiotics [5,20,86,123]. Skin testing and graded oral challenges should be reserved for patients in whom the implicated drug class is clinically important (e.g., β-lactams in pediatrics) and the initial SSLR was mild to moderate. In contrast, permanent avoidance is generally favored for severe reactions or when effective alternatives are readily available.

Contraindications to this approach include personal desire to avoid the risk of a distressful recurrence of SSLR (rare adverse effect), as well as pregnancy for GOCs [5,7,109].

The first step is skin testing (either patch or intradermal), given its safety profile; if positive, the patient must avoid the drug. If negative, the patient must undergo further testing with GOCs; a positive result indicates permanent hypersensitivity, while a negative result suggests that a future trial of this drug is safe. GOCs can be performed either in an outpatient setting or in an inpatient setting; it is recommended to perform GOCs in the hospital when the onset of SSLR is within 4–7 days after exposure to the causative agent [20,86]. The choice between shortened and prolonged GOCs remains controversial [5,20,83,86]. Most cases with a clinical diagnosis of SSLR test negative in both skin and oral challenge testing, suggesting transient hypersensitivity to the medication, the infectious agent, or their combination [41,126]. Skin testing seems to be safer than oral challenge testing but lacks sensitivity for non-IgE-mediated immunologic reactions [5,10,28,109,125,191]. The long-term management of SSLR is further complicated by the fact that its recurrence was noted in a significant portion of patients with a negative GOC [41]. Thus, clinicians should balance risk and benefit: permanent avoidance is generally favored after severe reactions, whereas tolerance testing may be appropriate in patients with mild prior SSLR and limited therapeutic alternatives.

Even if the final decision is to avoid the future administration of the causative agent, the decision regarding the safety of other drugs of the same class is less clear. For example, many authors believe that SSLR due to any β-lactam is a contraindication for the administration of other β-lactams [9,23]. On the contrary, other authors reject the importance of cross-reactivity in the development of β-lactam-induced SSLR, which is supported by a recent multicenter retrospective cohort study in Europe [9,23,36,47,52,84,86,87]. Moreover, 25 pediatric patients with a past medical history of β-lactam-induced SSLR underwent prolonged GOC with a different β-lactam, but only 1 patient developed mild urticaria [46]. A similar controversy exists in the literature about the future use of other tetracyclines in patients with minocycline-induced SSLR [23]. Taken together, the available evidence suggests that cross-reactivity among β-lactams in SSLR is likely lower than previously assumed, although not negligible. In clinical practice, this means that strict avoidance of all β-lactams may be unnecessarily restrictive, but tolerance testing should be carefully considered on a case-by-case basis, ideally in specialized allergy settings, to balance patient safety with antibiotic stewardship. Given the limited evidence in the available literature, further research is necessary to clarify the safety of administering similar drug agents.

Pretreatment with antihistamines and/or corticosteroids before the administration of a medication, e.g., monoclonal antibody, despite a previous episode of SSLR, remains controversial [51,68,101,105]. Premedication with NSAIDs in patients with a history of omalizumab-induced SSLR has also been proposed [138]. Patients with a positive history for transfusion-induced SSLR should receive plasma-free blood products in the future [158,192]. Successful desensitization has been reported, but its safety is controversial [10,39,109].

The recurrence of SSLR is disturbing for many patients who would prefer to avoid re-exposure to the same drug or a similar drug; discussion between the physician and the patient about the risks and the benefits is recommended [5,7,59,75]. These issues must be investigated by further clinical research.

## 11. Clinical Pearls

❖ Etiology: SSLR is most commonly triggered by β-lactam antibiotics, but monoclonal antibodies, vaccines, infections, and other drugs are increasingly recognized as potential causes.
❖ Clinical presentation: The classic triad includes fever, rash, and arthralgia, typically appearing 1–2 weeks after exposure. Complement levels are usually normal, and systemic organ involvement is rare, helping distinguish SSLR from classical SS.
❖ Diagnosis: Primarily clinical; laboratory investigations are useful to exclude differential diagnoses rather than confirm SSLR. Histopathology is reserved for uncertain cases.
❖ Treatment: First-line management is discontinuation of the offending agent. Mild cases respond to antihistamines and NSAIDs; moderate to severe cases may require systemic corticosteroids, with plasmapheresis reserved for refractory disease.
❖ Long-term management: Long-term management requires careful balance between permanent avoidance and tolerance testing, with skin testing and graded oral challenges reserved for mild cases involving essential drugs, while severe reactions generally warrant strict avoidance. Desensitization and premedication strategies remain experimental and should be undertaken cautiously.
❖ Prognosis: SSLR is generally self-limiting and resolves with supportive care, but recognition is crucial to prevent unnecessary interventions, guide safe future prescribing, and address patient anxiety regarding recurrence.

## 12. Conclusions

SSLR is a rare yet clinically important immunologic reaction that presents substantial diagnostic and therapeutic challenges due to its heterogeneous etiology and overlapping features with other conditions. Although typically benign and self-limiting, it can cause considerable morbidity and may lead to unnecessary investigations or inappropriate treatments if not promptly recognized. Diagnosis currently relies on clinical assessment, particularly recognition of the characteristic triad of rash, fever, and arthralgia, as no definitive laboratory markers exist, and available tests are used primarily to exclude alternative diagnoses. Management focuses on discontinuation of the offending agent and symptomatic therapy with antihistamines and NSAIDs for mild cases, reserving corticosteroids for severe presentations and plasmapheresis for refractory cases.

Critical uncertainties remain, including the safety of re-exposure to the same or related drugs, the lack of standardized diagnostic criteria, and the absence of reliable biomarkers for early detection or prognosis. The immunopathogenesis and risk factors of SSLR are still poorly understood, and predictors of severe or recurrent disease have not been clearly defined. Addressing these knowledge gaps through large-scale observational studies and rigorously designed clinical trials is imperative.

Beyond its clinical implications, SSLR also carries broader public health relevance. Fear of SSLR may influence antibiotic prescribing patterns, discourage the use of first-line β-lactams, and inadvertently contribute to antimicrobial resistance, while over-diagnosis can lead to unnecessary drug avoidance and restricted therapeutic options. Historical experience with cefaclor illustrates this dynamic: in a pediatric series, SSLR represented the most frequent adverse reaction to cefaclor (42%), and children with SSLR were significantly more likely to be treated with systemic corticosteroids in addition to antihistamines compared with those presenting with urticaria or erythema multiforme [193]. These findings may not only underscore the morbidity associated with SSLR but also help explain why concerns about recurrence have contributed to the decline in cefaclor prescribing in many countries.

More broadly, prescribing decisions are often shaped by clinical uncertainty and external pressures. In a qualitative study of hospital physicians in Norway, high workload, diagnostic ambiguity, limited infectious disease support, and patient or caregiver expectations were identified as key drivers of antibiotic prescribing behaviors [194]. While no study has specifically examined the association between fear of SSLR and antibiotic prescribing patterns, it is plausible that similar dynamics apply. Physicians’ concerns about SSLR, particularly in children, may lower their threshold for avoiding narrow-spectrum β-lactams and opting instead for second-line or broader-spectrum alternatives. This has important implications for antimicrobial prescription, as unnecessary avoidance of first-line agents may accelerate the development of resistance while limiting therapeutic options for future infections.

Similarly, isolated reports of SSLR following vaccinations, including influenza, pneumococcal, rabies, and COVID-19 vaccines, may fuel vaccine hesitancy despite the very low absolute risk and uncertain causality. This is particularly relevant given that vaccine hesitancy had a notable impact on the COVID-19 pandemic, with estimates suggesting that hesitancy and suboptimal interventions increased mortality risk up to 7.6-fold [195]. Even a modest 1% rise in hesitancy was predicted to reduce vaccine coverage by nearly 30% in India [196]. Surveys also reveal persistent hesitancy in many countries despite widespread availability, with levels as high as 48% in Russia and over 40% in Nigeria and Poland [197], while concerns about safety and side effects remain leading drivers across populations [198,199,200]. Healthcare workers are not exempt, with hesitancy reported in a minority of physicians and nurses, often linked to perceived vaccine risks [201,202]. The spread of misinformation through social media has amplified these fears, echoing historic anti-vaccine narratives that portray vaccines as more dangerous than the diseases they prevent [203,204,205,206]. Strengthening awareness that SSLR is uncommon, usually self-limited, and most often associated with favorable outcomes is essential to mitigate these unintended consequences. Ultimately, improved recognition, standardized diagnostic frameworks, and evidence-based management strategies will not only enhance patient care but also help preserve confidence in both antibiotic prescription and vaccination programs.

Lastly, considering future directions, priority should be given to large-scale, prospective studies that integrate clinical, immunologic, and pharmacogenomic data to identify biomarkers of susceptibility and prognosis. Equally important are studies that evaluate communication strategies to reduce unnecessary drug avoidance and vaccine hesitancy, ensuring that advances in understanding SSLR translate into both improved patient care and strengthened public health confidence.

## Figures and Tables

**Table 1:** Key differences between SSLR and SS.

	SSLR	SS
Causes	Viruses, bacteria, vaccines, and small non-protein drugs such as antibiotics. There is controversy about the monoclonal antibodies in the literature.	Proteins of high molecular weight in heterologous serum, such as antirabies serum. There is controversy about the monoclonal antibodies in the literature.
Pathogenesis	Unknown; proposed mechanisms involve altered drug metabolism, haptens, lymphotoxicity, IgE/eosinophils, neutrophils, ADAs, type III hypersensitivity reaction	Type III hypersensitivity reaction (deposition of immune complexes and complement activation)
Dose/frequency	Probably an idiosyncratic reaction.	Larger doses and repeated exposure are more likely to trigger SS.
Clinical presentation	The involvement of lymph nodes or internal organs is unlikely.	Lymphadenopathy, hepatitis, renal involvement, serositis, pneumonitis, and neurological complications are more likely to occur in SS than SSLR, even though these complications are rare.
Age	Usually children.	Usually adults.
Laboratory testing	Complement is usually normal.	Complement is usually lower than normal.
Histopathology	SSLR is included in urticaria spectrum disorders. Skin biopsy typically reveals a perivascular and interstitial infiltrate of neutrophils, lymphocytes, and eosinophils. Neutrophils are the dominant cells. The presence of leukocytoclastic vasculitis and the deposition of immune complexes or complement is unusual.	Leukocytoclastic vasculitis. Direct immunofluorescence reveals the deposition of ICs and complement.

Note: SSLR: Serum sickness-like reaction; SS: serum sickness; ADAs: anti-drug antibodies; ICs: immune complexes.

**Table 2:** Classification of Causative Agents in SSLR.

Category Agent	Examples		
Antimicrobials	B-lactams		penicillin V *, penicillin G *, carbenicillin, ciclacillin, flucloxacillin, cloxacillin, amoxicillin *, amoxicillin/clavulanate *, piperacillin/tazobactam, meropenem *, cephalexin *, cefazolin *, cefprozil *, cefdinir, cefaclor *, cefuroxime *, ceftriaxone *, loracarbef *, ceftazidime *, cefixime *, cephalothin, cefditoren, cefdinir *
	Macrolides		Erythromycin *, azithromycin, clarithromycin
	Quinolones		moxifloxacin, levofloxacin, ciprofloxacin
	Antitubercular		isoniazid, rifampicin, para-aminosalicylic acid
	Other antibiotics		minocycline *, metronidazole, trimethoprim/sulfamethoxazole *, vancomycin, streptomycin, furazolidone *, nafcillin
	Antifungals		itraconazole, griseofulvin, terbinafine
	Antivirals		Valacyclovir, nevirapine *
	Anthelmintic		Levamizole *
Monoclonal antibodies		Infliximab *, rituximab *, omalizumab, adalimumab *, dupilumab, efalizumab, etanercept *, certolizumab *, eculizumab, ixekizumab, secukinumab, alemtuzumab, ustekinumab, vedolizumab, mepolizumab, natalizumab, dalotuzumab, ocrelizumab, trastuzumab	
Anti-inflammatory		acetaminophen *, ibuprofen, naproxen, propyphenazone, phenylbutazone, salicylates, prednisone, prednisolone, methylprednisolone	
Infectious agents	Bacteria		Streptococcus *, Mycoplasma *
	Viruses		Adenovirus *, influenza, EBV *, CMV *, HHV-6 *, HBV *, NANBH
Vaccines		rabies *, influenza *, tetanus toxoid, HBV, pneumococcal vaccines, MMR, COVID-19 vaccines	
Immunosuppressants/ Antineoplastic		bendamustine, cyclosporine, MMF, fludarabine, tacrolimus, methotrexate, azathioprine, cyclophosphamide, lenalidomide, sirolimus-eluting stent, dalotuzumab/MK-2206 *	
Psychiatric medications		bupropion *, fluoxetine, paroxetine, risperidone, phenobarbital, etifoxine *	
Anticonvulsants		carbamazepine *, phenytoin	
Bronchodilators		albuterol, montelukast	
Cardiovascular drugs		hydralazine, propranolol, ivabradine *, captopril, streptokinase *, clopidogrel, ticlopidine, pamabrom	
Endocrine drugs		thiouracil, insulin, thyroid preparations,	
Others		blood products, allopurinol, ondansetron, mirabegron, zoledronic acid, lansoprazole, filgrastim, cetirizine, elezacaftor/tezacaftor/ivacaftor, D-mannose (supplement), drotaverine, mesalamine, cholecystographic dyes, CITC-DTPA, insect venom, iron dextran, ferric carboxymaltose, insect sting *	

Note: (*) Evidence derived from studies other than single case reports, including basic research, observational cohorts, and systematic reviews. HBV: hepatitis B virus; MMR: measles–mumps–rubella; COVID-19: coronavirus disease 2019; MMF: mycophenolate mofetil; NANBH: non-A, non-B hepatitis; EBV: Epstein–Barr virus; CMV: Cytomegalovirus; HHV-6: Human Herpesvirus 6.

**Table 3:** Specific incidence of SSLR after the administration of certain drugs.

Cefaclor	Heckbert et al. [69]: 0.14%,
	Levine [73]: 0.2% (Up to 0.5% with Multiple Courses),
	Hyslop [118]: 0.024%,
Amoxicillin	Heckbert et al. [69]: 0.0074%,
TMP-SMZ	Heckbert et al. [69]: 0.089%,
penicillin V	Heckbert et al. [69]: 0.043%,
Ampicillin	Caldwell, Cluff [119]: 0.5% (twice in pediatric patients under 10 years),
Nafcillin	Blumenthal et al. [59]: 0.214%
IFX	Hanauer et al. [93]: 2.44%,
	Hamzaoglu et al. [60]: 0.3%
	Seiderer et al. [75]: 1%,
	Colombel et al. [68]: 2.8%,
	Teshima et al. [66]: 3.42%
	Lew et al. [65]: 4%
	Kugathasan et al. [71]: 9%,
Anti-TNF	Abraham et al. [67]: 2.5%,
RTX	Gottenberg et al. [94]: 2.44% (primary SS)
	Godeau et al. [76]: 1.67% (ITP),
	Isaksen et al. [70]: 8% (primary SS),
Omalizumab	Molderings et al. [72]: 0.4–0.6% (up to 25% in the presence of mast cell activation disorders),
HDCRV	Warrington et al. [74]: 2.06%
GPO-MBP inactivated influenza vaccine	Apisarnthanarak et al. [61]: 3%
Bupropion	Beyens et al. [62]: 0.002% (yearly incidence)
Streptokinase	Lee et al. [63]: 1.754%
	Bucknall et al. [120]: 2.5%,

Note: IFX: Infliximab; RTX: Rituximab; HDCRV: Human diploid cell rabies vaccine; TMP-SMZ: Minocycline and trimethoprim-sulfamethoxazole; GPO-MBP: Government Pharmaceutical Organization-Mérieux Biological Products; TNF: Tumor necrosis factor.

**Table 4:** Key Differences between pediatric and adult SSLR.

	Children	Adults
Epidemiology	More common in children (particularly under 10 years)	Adults are affected less often, with different trigger profiles
Triggers	Most often β-lactam antibiotics (esp. amoxicillin, cefaclor)	Monoclonal antibodies, antidepressants, TMP–SMZ, cephalexin
Diagnosis	Frequently misdiagnosed; only ~30% initially recognized; hospitalization is often driven by diagnostic uncertainty (e.g., to exclude septic arthritis or severe hypersensitivity)	Diagnosis generally more straightforward, with fewer hospital admissions
Management	Corticosteroids are often prescribed, but evidence of benefit is weak; need to balance against growth-related and systemic risks	Similar use of corticosteroids, but risk–benefit assessment differs as concerns about growth and developmental side effects are less relevant
Clinical Implication	Pediatric SSLR requires heightened awareness, cautious use of corticosteroids, and diagnostic strategies tailored to distinguish it from mimics	Adult management is generally less complex, though agent-specific risks still need consideration

**Table 5:** Variation in Reported Frequencies of Fever, Rash, and Joint Involvement in SSLR by Study Design, Population, and Definition.

Authors	Triggers	Fever	Rash	Arthralgia/Arthritis
Del Pozzo-Magaña et al. [7]	mostly antibiotics (especially β-lactams)	44.5%	100% (inclusion criteria)	100% (inclusion criteria)
Delli Coli et al. [19]	antibiotics (especially β-lactams)	40%	n/a	100%
Mohsenzadeh et al. [45]	mostly antibiotics (especially β-lactams)	45%	98%	91.5%
Yorulmaz et al. [13]	mostly antibiotics	41.4%	89.7%	82.8%
Karmacharya et al. [127]	rituximab	78.8%	69.7%	72.7%
Eichenfield et al. [75]	minocycline	79%	100%	100%
Stricker et al. [126]	Cefaclor	10%	70.2%	56.2%
	Amoxicillin	5.9%	88.2%	100%
	Cephalexin	16.7%	100%	100%
Friedman et al. [4]	antibiotics (especially β-lactams)	39.3%	97.8%	84.3%
Khalaf et al. [6]	mostly antibiotics (especially β-lactams)	35.1% (children)	100% (children)	88.2% (children)
		72.3% (adults)	100% (adults)	84.1% (adults)

**Table 6:** The differential diagnosis of SSLR.

Infectious Diseases	
Bacterial infections	Lyme disease, infective endocarditis, septic arthritis, syphilis, disseminated gonococcal infection, rickettsial disease, cat scratch disease, rat-bite fever, Brucella, meningococcemia, scarlet fever (Streptococcus pyogenes)
Viral infections	HAV, HBV, HCV, HIV, arbovirus infection, infectious mononucleosis, CMV, rubella, coxsackie, parvovirus B19, enterovirus, and coronavirus.
Fungal infections	Histoplasmosis, Blastomycosis, Coccidiomycosis, Aspergillus, angioinvasive soft tissue infections
Neoplastic diseases	leukemia, lymphoma, plasma cell disorders, mast cell disorders
Eosinophilic disorders	eosinophilic cellulitis, eosinophilic annular erythema, eosinophilia-myalgia syndrome, drug reaction with eosinophilia and systemic symptoms
Urticaria spectrum disorders	Anaphylaxis, urticaria, UM, and UV
Transfusion reactions	(a) acute febrile non-hemolytic reactions, allergic reactions (b) Immediate infusion reaction to monoclonal antibodies
Rheumatic diseases	RA, SLE, drug-induced lupus, JIA, ARF, sarcoidosis, contaminated heroin-induced vasculitis, reactive arthritis, gout, pseudogout, Kawasaki, HSP, adult-onset Still’s disease, CAPS, TRAPS, Schnitzler syndrome, PFAPA, PAN, FMF, AHEI, cryoglobulinemia, dermatomyositis, and scleroderma
Bullous dermatoses	PV, BP, linear IgA disease
Other	EM major or minor, SJS/TEN, AGEP, acneiform eruption, exanthematous drug eruption, exfoliative dermatitis, GVHD, SS, hypersensitivity angiitis

Note: UM: acute annular erythema/urticaria multiforme; UV: urticarial vasculitis; HAV: hepatitis A virus; HBV: hepatitis B virus; HCV: hepatitis C virus; HIV: human immunodeficiency virus; CMV: cytomegalovirus; RA: rheumatoid arthritis; SLE: systemic lupus erythematosus; JIA: juvenile idiopathic arthritis; ARF: acute rheumatic fever; HSP: Henoch–Schönlein purpura; CAPS: cryopyrin-associated periodic syndrome; TRAPS: TNF-receptor associated periodic syndrome; PFAPA: periodic fever–aphthous stomatitis–pharyngitis–adenitis syndrome; PAN: polyarteritis nodosa; FMF: familial Mediterranean fever; AHEI: acute hemorrhagic edema of infancy; PV: pemphigus vulgaris; bullous; ΒΡ: pemphigoid; SSLR: Serum sickness-like reaction; ΕΜ: erythema multiforme; SJS/TEN: Stevens Johnson syndrome/Toxic Epidermal Necrolysis; AGEP: acute generalized erythematous pustulosis; IgA: Immunoglobulin A; GVHD: graft-versus-host-disease; SS: serum sickness.

## Data Availability

No new data were created or analyzed in this study. Data sharing is not applicable to this article.

## Abbreviations

The following abbreviations are used in this manuscript:

ADA	Anti-drug antibodies
AGEP	Acute generalized erythematous pustulosis
ALG	Antilymphocyte globulin
ANA	Antinuclear antibodies
ARDS	Acute respiratory distress syndrome
ATG	Antithymocyte globulin
BP	Bullous pemphigoid
CAPS	Cryopyrin-associated periodic syndrome
CBC	Complete blood count
CCP	Cyclic citrullinated peptide
CMV	Cytomegalovirus
COVID-19	Coronavirus disease 2019
CRP	C-reactive protein
DIF	Direct immunofluorescence
DRESS	Drug reaction with eosinophilia and systemic symptoms
EM	Erythema multiforme
ESR	Erythrocyte sedimentation rate
FDA	Food and Drug Administration
FMF	Familial Mediterranean fever
GOCs	Graded oral challenges
GVHD	Graft-versus-host disease
HBV	Hepatitis B virus
HACA	Human antichimeric antibodies
HAV	Hepatitis A virus
HCV	Hepatitis C virus
HDCRV	Human diploid cell rabies vaccine
HSP	Henoch–Schönlein purpura
HSV	Herpes simplex virus
IC	Immune complex
IgE	Immunoglobulin E
IgG	Immunoglobulin G
IgM	Immunoglobulin M
IL-6	Interleukin-6
ITP	Immune thrombocytopenic purpura
IV	Intravenous
JIA	Juvenile idiopathic arthritis
LDH	Lactate dehydrogenase
LFT	Liver function test
LTA	Lymphocyte toxicity assay
LTT/LST	Lymphocyte transformation/stimulation test
MAS	Macrophage activation syndrome
MMF	Mycophenolate mofetil
MMR	Measles–mumps–rubella
MRI	Magnetic resonance imaging
NETs	Neutrophil extracellular trap
NSAID	Non-steroidal anti-inflammatory drug
NUD	Neutrophilic urticarial dermatosis
PAN	Polyarteritis nodosa
PFAPA	Periodic fever–aphthous stomatitis–pharyngitis–adenitis syndrome
PV	Pemphigus vulgaris
RA	Rheumatoid arthritis
RAST	Radioallergosorbent test
PCT	Procalcitonin
PSP	Pancreatic stone protein
RTX	Rituximab
SJS/TEN	Stevens–Johnson syndrome/Toxic epidermal necrolysis
SLE	Systemic lupus erythematosus
SoJIA	Systemic-onset juvenile idiopathic arthritis
SS	Serum sickness
SSLR	Serum sickness-like reaction
TMP–SMZ	Trimethoprim–sulfamethoxazole
TNF	Tumor necrosis factor
TRAPS	TNF-receptor associated periodic syndrome
UM	Urticaria multiforme
UV	Urticarial vasculitis
WBCs	White blood cells
